# Pregnant Sheep in a Farm Environment Did Not Develop Anaemia

**DOI:** 10.3390/ani7050034

**Published:** 2017-04-25

**Authors:** Gabrielle C. Musk, Amanda James, Matthew W. Kemp, Sara Ritchie, Andrew Ritchie, Michael Laurence

**Affiliations:** 1 Animal Care Services, University of Western Australia, Crawley 6009 Australia; 2 College of Veterinary Medicine, Murdoch University, Murdoch 6150, Australia; ajames722@hotmail.com (A.J.); m.laurence@murdoch.edu.au (M.L.); 3 School of Women’s and Infants’ Health, University of Western Australia, Crawley 6009, Australia; matthew.kemp@uwa.edu.au; 4 Icon Agriculture, Darkan 6392, Australia; sara@iconag.com.au (S.R.); andrew@iconag.com.au (A.R.)

**Keywords:** anaemia, pregnancy, sheep

## Abstract

**Simple Summary:**

Anaemia refers to a low red blood cell count and is common during pregnancy in women. Anaemia has been reported in sheep undergoing surgery during pregnancy for biomedical research projects. The incidence and severity of anaemia during pregnancy in sheep is unknown. Three groups of sheep were established: non-pregnant; pregnant with a single lamb; and pregnant with twin lambs. The stage of pregnancy was known as the sheep were mated on a known date and pregnancy was confirmed 50 days later by ultrasound examination. During pregnancy, blood samples were collected to measure red cells in the blood and protein levels. Samples were also collected to ensure that the sheep did not have any risk factors for anaemia, such as nutritional deficiency and internal parasites. Anaemia did not occur in any sheep during the study. Anaemia did not develop during pregnancy and red cell counts and protein levels were similar between each group. All the sheep lambed as expected, except for one that was expecting twins delivered a single lamb.

**Abstract:**

The aim of this study was to document the haematological profile of pregnant ewes throughout gestation. Sheep were divided into three groups (*n* = 8 per group): non-pregnant, singleton, or twin pregnancy. Blood samples were collected every 14 days from day 55 of gestation for haemoglobin concentration; packed cell volume; total protein; and albumin concentration. On days 55 and 125 of gestation blood was collected for trace element estimation: soluble copper and zinc; glutathione peroxidase (GSHPx); and methylmalonic acid (MMA). Pooled faecal samples were collected on days 55, 97, and 139 of gestation. Pasture cuts were collected on days 97 and 153 of gestation. The haematology and protein concentrations were not different between groups throughout the study. Copper concentration increased in all animals during the study (*p* < 0.0001). Zinc concentration was lowest in the singleton and twin pregnant sheep on day 55 of gestation (*p* = 0.04). GSHPx was not different between groups during the study. MMA decreased in all animals during the study (*p* < 0.0001), but was not different between groups. Faecal samples were consistently negative for strongyle and nematode eggs, and coccidian oocysts. The pasture was good quality. Pregnant sheep in a farm environment with normal trace element status, no parasites, and an adequate diet, did not develop anaemia (PCV < 0.27).

## 1. Introduction

Anaemia commonly occurs in pregnant women in the third trimester and affects nearly half of the world’s population of pregnant women [1,2]. Approximately 75% of these cases are attributed to iron deficiency, while a large proportion of the remaining cases are due to folic acid or vitamin B12 deficiencies [1,2]. Physiological anaemia also occurs during late pregnancy when there is a disproportionate increase in plasma volume compared to red cell mass in an attempt to improve oxygenation within the body, promote growth of the fetus, and prepare for hemostasis during delivery [1,2]. In conjunction with this physiological alteration, there is a decrease in haemoglobin concentration in the body which can be associated with premature births, low birth weights, or abortions in severely affected women [2]. 

While anaemia during pregnancy has been well-documented in humans, there are limited data about this abnormality in pregnant sheep. In a recent study of twin-bearing ewes, arterial blood samples collected during general anaesthesia demonstrated maternal anaemia (low packed cell volume and haemoglobin concentration) [3]. Likewise, a similar study in singleton ewes described maternal anaemia during general anaesthesia [4]. The cause of this anaemia may have been the administration of drugs that alter vascular tone and the administration of intravenous fluid therapy, which alters vascular volume [4,5]. Although it is possible that this anaemia was a consequence of these iatrogenic alterations, there is limited information about the development of anaemia during pregnancy in sheep to verify this hypothesis. Given the extensive use of pregnant sheep for biomedical research projects investigating the causes and consequences of preterm birth [6] it is essential to ensure that the physiological features of the model are thoroughly understood.

Anaemia in sheep most commonly occurs after trauma, gastrointestinal or internal parasitism (e.g., *Haemonchus contortus* or *Mycoplasma ovis*), tick infestation, ingestion of toxic plants (such as onion, kale and rapeseed), immune-mediated or chronic diseases, or the presence of bacterial toxins (in particular *Clostridium haemolyticum* and *Clostridium perfringens* type A) [7]. Trace element deficiencies, such as copper and selenium, may also cause Heinz body anaemia in sheep [7].

The aim of this study was to document the haematological profile of pregnant ewes throughout gestation to determine when, and to what extent, anaemia develops in these sheep. The hypothesis was that sheep would develop moderate to severe anaemia at the end of the second trimester of gestation and that the anaemia would be more severe in twin-bearing ewes than singleton ewes. 

## 2. Materials and Methods

The study was approved by the Animal Ethics Committees of the University of Western Australia and Murdoch University (RA.3.100.1452), in accordance with the Code of Practice for the Care and Use of Animals for Scientific Purposes [8]. 

Twenty-four Merino ewes on a rural property in Darkan, Western Australia (33°18′54.6′′ S, 116°42′14.2′′ E) were enrolled in the study in April (autumn). The sheep were induced to ovulate (300 mg progesterone intravaginal, Eazi-Breed Sheep and Goat Device, Zoetis, NJ, USA) and naturally mated on a known date (within a 24 h window). Pregnancy diagnosis was confirmed 50 days later by ultrasound examination. Based on the ultrasound diagnosis, sheep were divided into one of three groups (*n* = 8 per group): non-pregnant; singleton pregnancy; or twin pregnancy. 

The six-year-old sheep were administered a broad-spectrum anthelmintic drench (Abamectin 0.8 g/L and Selenium 0.4 g/L, Abaguard Plus Selenium, Dalgety Animal Health, Landmark Operations, Dandenong, Australia) prior to mating. Prior to lambing, the sheep were vaccinated against cheesy gland and five Clostridial diseases (Cydectin Eweguard SE B12 6 in 1 Vaccine and Wormer, Virbac Australia Pty. Ltd., Milperra, Australia). The sheep were fed oats, starting at 100 g/head/day and increasing to 200 g/head/day immediately prior to mating. After mating, they were fed 200 g/head/day of lupins with approximately 40 g/head/day pellets (Macco Feeds 707 pellets). The pellets were formulated to meet the requirements for growth in prime lambs and contain 16% protein in dry matter, 12 MJ of metabolisable energy/kg of dry matter, four times the recommended minimum level of Cobalt and Selenium and three times the recommended minimum level of Zinc (personal communication John Milton). Supplemental feeding was stopped in June (~85 days of gestation) because the pasture appeared adequate. The sheep remained in their standard farm environment for the duration of the study.

Blood samples were collected from the jugular vein, and stored in EDTA anticoagulant, every 14 days (on days 55, 69, 83, 97, 111, 125, and 139) for selected haematology and biochemistry analyses: haemoglobin concentration (Hb); packed cell volume (PCV); total protein (TP) concentration; and albumin concentration. An additional blood sample was collected within three days of parturition for assessment of the same parameters. On days 55 and 125 of gestation, an additional sample of blood was collected and stored in lithium heparin anticoagulant for trace element analyses and estimation: soluble copper and zinc; glutathione peroxidase (GSHPx); and methylmalonic acid (MMA). Sheep were restrained in the standing position by a skilled sheep handler and their necks were extended to facilitate blood collection. Haematology and biochemistry analyses were performed by a commercial veterinary pathology laboratory (VetPath Laboratory Services, Ascot, Western Australia) and trace element analyses were performed by the government laboratory (Department of Agriculture and Food, South Perth, Western Australia).

Pooled faecal samples were collected from the yards following blood collection on days 55, 97, and 139 of gestation. Faecal egg counts were performed by the government laboratory (Department of Agriculture and Food, South Perth, Western Australia), to assess parasite burden.

Pasture cuts were collected on days 97 and 153 of gestation. Several one square meter areas of pasture were cut using hand shears to ground level to reflect the food on offer available to the sheep. The pasture was sealed in zip lock bags with as much air removed as possible. The pasture was analysed by a commercial laboratory (Agrifood Technology, FeedTest, Werribee, Australia) to yield information about nutritional content (dry matter, moisture, ash, crude protein, digestibility, metabolisable energy, neutral detergent fibre and acid detergent fibre).

Data are presented as mean (SD). Data were tested for normality and compared with analysis of variance or a *t*-test (GraphPad Prism 7, GraphPad Software Inc., La Jolla, CA, USA). Statistical significance was set at *p* < 0.05.

## 3. Results

All sheep delivered their lambs between 147 and 151 days of gestation and consistently had a body condition score of 2.5–3 (scale 0–5). No sheep determined to be non-pregnant delivered any lambs. All sheep in the singleton group delivered one lamb. One sheep in the twin pregnancy group only delivered one lamb.

**Figure 1:**
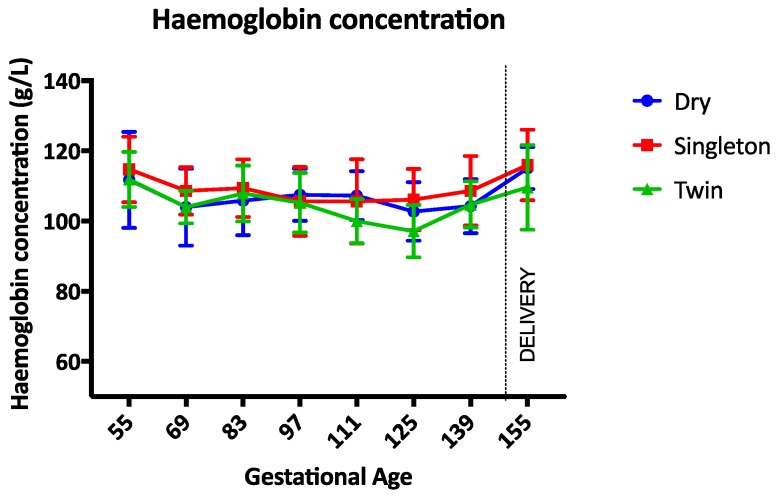
Mean (SD) haemoglobin concentration during the study period. Circle = dry ewes, Square = singleton pregnant ewes, Triangle = twin pregnancy ewes.

**Figure 2:**
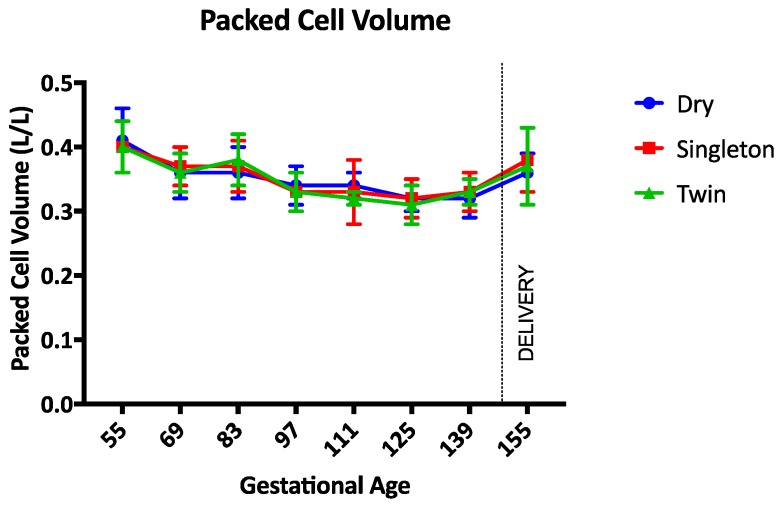
Mean (SD) packed cell volume during the study period. Circle = dry ewes, square = singleton pregnant ewes, triangle = twin pregnancy ewes.

**Figure 3:**
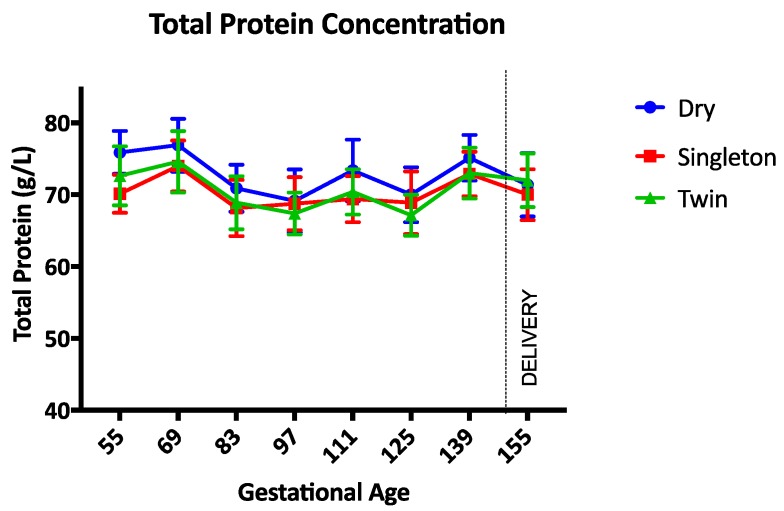
Mean (SD) total protein concentration during the study period. Circle = dry ewes, square = singleton pregnant ewes, triangle = twin pregnancy ewes.

**Figure 4:**
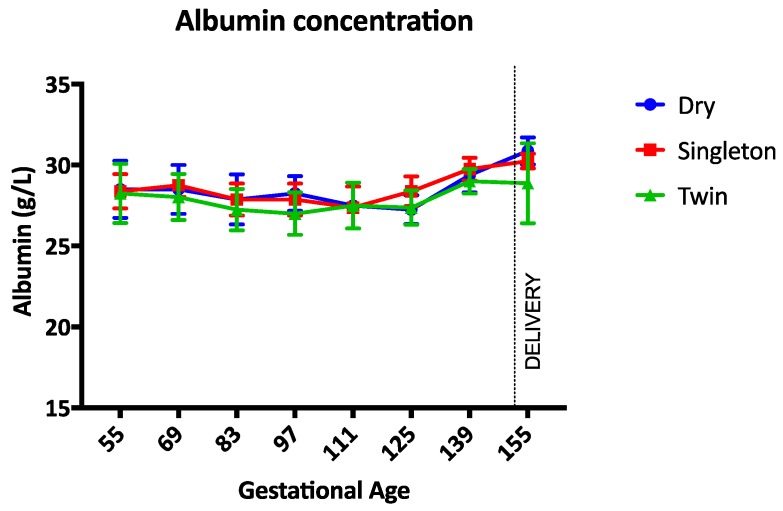
Mean (SD) albumin concentration during the study period. Circle = dry ewes, square = singleton pregnant ewes, triangle = twin pregnancy ewes.

The haemoglobin concentration was not different between groups during the study (Figure 1). Between gestational days 111 and 125 the haemoglobin concentration was lowest in twin lambs but this difference was not statistically significant (*p* = 0.17). The PCV, TP, and albumin concentrations were not different between groups throughout the study period (*p* = 0.97, *p* = 0.14, and *p* = 0.34 respectively) (Figure 2, Figure 3 and Figure 4). Haemoglobin, PCV, and TP remained within the reference intervals provided by the veterinary pathology laboratory, while the albumin concentration was below the reference interval for the duration of the study. The reference intervals were: Hb 90–150 g/L; PCV 0.27–0.45; TP 60–82 g/L; and albumin 30–40 g/L (VetPath Laboratory Services, Ascot, Western Australia).

Trace element analyses revealed a significant increase in copper concentration in all animals during the study (*p* < 0.0001) but no difference between groups (Table 1). The concentration of zinc was lowest in the singleton and twin pregnant sheep on day 55 of gestation (*p* = 0.04). There was no difference in the concentration of GSHPx between groups during the study. The concentration of MMA decreased in all animals during the study, but was not different between groups (*p* < 0.0001) (Table 1).

Pooled faecal samples were consistently negative for strongyle and nematode eggs, and coccidian oocysts. The results of analysis of the first pasture sample suggest that the sample was not cleaned adequately of soil prior to analysis, as there is a very low digestibility, low metabolisable energy, and high ash content. The subsequent sample indicates that the pasture was good quality (Table 2).

## 4. Discussion

This study was performed to determine if anaemia develops during pregnancy in sheep. The results of the study are to be applied to the biomedical research context where pregnant sheep may undergo general anaesthesia for surgery and fetal intervention. The sheep in this study did not develop anaemia during gestation so the hypothesis that anaemia of pregnancy causes intra-operative anaemia, as previously reported, was not proved. 

There was no difference, or abnormality, in the haemoglobin, PCV or TP concentrations in the sheep in this study. Pregnancy status, season, and trace element status did not cause anaemia or hypoproteinaemia. The exception to this statement is the albumin concentration, which was lower than the low end of the reference range in all sheep. Given that all the sheep had similar concentrations of albumin and the total protein concentration was normal, the significance of this finding is unclear. There are no universally-accepted reference values and ranges for clinical biochemistry parameters in sheep [9]. Furthermore, there are many different sheep breeds and laboratory techniques so reference ranges for these parameters should be developed specifically for each laboratory. The albumin concentrations in this study were only slightly below the lower end of the reference range, so it is possible that the total protein concentration was normal because of an increase in globulins [9,10]. 

Albumin can decrease in sheep for a variety of reasons including internal parasitism, infiltrative bowel disease or liver disease [11]. With the limited haematological and biochemical tests performed in this study, there was no evidence of parasitism, bowel, or liver disease in the sheep in this study. Albumin can also decrease if the sheep has a protein maldigestion or malabsorption disorder [11]. While albumin was low, total protein values were normal in all sheep making these disorders less likely. Further investigations such as urinalysis and urine protein:creatinine ratio could be performed to further explore the cause of the hypoalbuminaemia [11].

In previous studies in twin pregnant ewes during general anaesthesia, the maternal PCV and Hb were 19.7 (±3.4) and 67.4 (±11.7) g/L respectively [3] and in singleton ewes during anaesthesia these parameters were similarly low [4]. Plasma protein concentration was not measured in either study. The authors of these studies hypothesised that anaemia of pregnancy contributed to intra-operative anaemia but the results of this current study do not support that theory. It is more likely that drugs administered for anaesthesia caused a decrease in the PCV [12,13]. Furthermore, fluid therapy is commonly administered during anaesthesia to offset the negative effects of anaesthetic drugs and replace body fluids lost during surgery [14]. The aim of fluid therapy is to expand the vascular volume to improve cardiac output and perfusion [14]. By expanding the vascular volume, a dilutional anaemia is produced, which may have contributed to the anaemia in the sheep undergoing anaesthesia [15].

The trace element status of the sheep in this study was described by measuring circulating copper, zinc, GSHPx, and MMA on two occasions. Neither a trace element deficiency, nor toxicity was apparent because all the measured variables remained within the reference intervals for the duration of the study [16]. Copper has been reported to increase during pregnancy [17] but the copper concentration in the current study increased to a significant, and similar, extent in dry and pregnant sheep so it is more likely that season and/or nutritional status influenced this change. There are conflicting reports on the influence of season on copper levels in sheep. Yokus et al. (2004) conclude that the disparate results are a result of differences of breed, sex, and regional feeding practices between studies. The concentration of zinc was lower in the pregnant animals in early gestation but there was no such difference in late gestation. Zinc concentrations in pregnant sheep have previously been reported to be stable during pregnancy so the significance of this result in the current study is unknown [17]. Given that the sheep were not anaemic and appeared to progress through pregnancy normally, the lower concentration of zinc in pregnant sheep in early gestation is not likely to be clinically significant. Serum GSHPx was measured as an indicator of selenium deficiency as there is a high correlation between GSHPx and selenium status [18]. Selenium is essential for health and productivity in all animal species but, in ruminants, a deficiency in this element is associated with a range of disorders including reduced fertility and an increased rate and severity of infectious diseases [18]. The GSHPx concentrations in this study were not different between groups and were adequate. Selenium deficiency was unlikely in this study given that selenium supplementation was provided when the animals were vaccinated prior to mating and the soil in the region where the study was performed is at a low risk of deficiency in this element. Finally, MMA was measured to indicate cobalt and vitamin B12 status in these sheep. This compound will increase if cobalt and vitamin B12 are deficient [19]. The decrease over time in MMA in all groups in this study suggests that cobalt and vitamin B12 levels were adequate, as expected, given the vitamin B12 supplementation included with the vaccination.

The most prominent gastrointestinal parasite causing anaemia and hypoproteinaemia in sheep in many parts of the world is *Haemonchus contortus* [20], a nematode that feeds on blood and undergoes a period of arrested development (hypobiosis) in the abomasum, resulting in ineffective anthelmintic drenching if not timed appropriately [21]. As with many other parasites *Haemonchus contortus* has become resistant to many of the most common broad-spectrum anthelmintic drugs, such as the benzimidazoles, tetrahyrdopyrimidines, and macrocyclic lactones due to frequent dosing and widespread usage [21]. In this study, the faecal egg counts revealed that there were no nematode, strongyle, or coccidian eggs present in the faeces, indicating that the prophylactic drench was effective and that gastrointestinal parasites were successfully managed.

Analysis of a cut of pasture was performed to determine if the nutritional requirements of pregnancy were met. Unfortunately, the results of the first pasture sample indicate that the sample was not cleaned adequately of soil prior to analysis, as there is a very low digestibility, low metabolisable energy and high ash content. The subsequent sample, albeit from a different season (end of winter) indicates that the pasture was good quality with the metabolisable energy being in the good to excellent category [22,23]. Twin bearing ewes at medium body condition, similar to those in this study, can be offered restricted feeding with few implications for their lambs [23]. In the current study, the nutritional quality of the pasture should not have had adverse effects on the lamb [23], and we have shown that the consequences for the ewe are also minimal. Although extensive research has been undertaken to investigate the optimal nutritional status for twin pregnant ewes [23,24,25] the focus has been on the newborn lamb as an indication of maternal wellbeing. Although there were no differences in the parameters measured in the current study, a larger cohort of animals may be required to demonstrate a difference between the maternal alterations during pregnancy with single or multiple fetuses. 

## 5. Conclusions 

Pregnant sheep in a farm environment with normal trace element status, no evidence of parasitic infestation, and an adequate diet, did not develop anaemia. As pregnant sheep are commonly used as models for biomedical research, further investigations into the impact of anaesthesia and surgery on the development of anaemia are indicated to elucidate the impact of these interventions on the physiological health of the ewe and fetus.

## Figures and Tables

**Table 1:** The mean (SD) trace element concentrations on days 55 and 125 of gestation. Cu = copper, Zn = Zinc, GSHPx = glutathione peroxidase, MMA = methylmalonic acid. ** *p* < 0.0001 day 125 compared to day 55, * *p* = 0.04 singleton and twin compared to dry on day 55.

Trace Element Parameter	Cu (mg/mL)		Zn (mg/mL)		GSHPx (U/g/Hb)		MMA (μmol/L)	
Reference Interval ^1^	0.6–1.1		0.6–1.0		>50		<3	
Sample Collection	Day 55	Day 125	Day 55	Day 125	Day 55	Day 125	Day 55	Day 125
**Dry**	0.88 (0.15)	1.12 ** (0.25)	1.09 (0.15)	0.99 (0.07)	240.13 (48.6)	247.38 (48.9)	0.91 (0.36)	0.52 ** (0.19)
**Singleton**	0.75 (0.2)	1.04 ** (0.27)	0.90 * (0.12)	1.04 (0.15)	243.0 (55.3)	223.63 (50.8)	0.94 (0.18)	0.69 ** (0.27)
**Twin**	0.78 (0.22)	1.05 ** (0.2)	0.91 * (0.18)	0.97 (0.12)	261.8 (35.8)	245.50 (44.3)	1.04 (0.39)	0.57 ** (0.14)

**^1^** Reference intervals provided by Department of Agriculture and Food, Western Australia.

**Table 2:** Pasture cut analyses on days 97 and 153 of gestation.

Pasture Analysis Parameters	Pasture Cut Date	
	Day 97	Day 153
Dry matter (DM) (%)	33.3	18.9
Moisture (%)	66.7	81.1
Crude protein (% of DM)	8.2	15.5
Acid detergent fibre (% of DM)	61.8	21.1
Neutral detergent fibre (% of DM)	77.3	44.4
Digestibility (DMD) (% of DM)	25.6	77.5
Digestibility (DOMD) (% of DM)	28.5	72.5
Est. metabolisable energy (calculated **^1^**; MJ/kg DM)	2.8	11.7
Fat (% of DM)	-	3.7
Ash (% of DM)	56.2	8.7

**^1^** Reference intervals provided by Department of Agriculture and Food, Western Australia.
